# Light-Induced Transformation of Virus-Like Particles on TiO_2_

**DOI:** 10.1021/acsami.4c07151

**Published:** 2024-07-03

**Authors:** Mona Kohantorabi, Aldo Ugolotti, Benedikt Sochor, Johannes Roessler, Michael Wagstaffe, Alexander Meinhardt, E. Erik Beck, Daniel Silvan Dolling, Miguel Blanco Garcia, Marcus Creutzburg, Thomas F. Keller, Matthias Schwartzkopf, Sarathlal Koyiloth Vayalil, Roland Thuenauer, Gabriela Guédez, Christian Löw, Gregor Ebert, Ulrike Protzer, Wolfgang Hammerschmidt, Reinhard Zeidler, Stephan V. Roth, Cristiana Di Valentin, Andreas Stierle, Heshmat Noei

**Affiliations:** † Centre for X-ray and Nano Science CXNS, Deutsches Elektronen-Synchrotron DESY, 22607 Hamburg, Germany; ‡ Dipartimento di Scienza dei Materiali, Università degli Studi di Milano-Bicocca, Via Cozzi 55, 20125 Milano, Italy; § Deutsches Elektronen-Synchrotron DESY, Notkestr. 85, 22607 Hamburg, Germany; ∥ Advanced Light Source, Lawrence Berkeley National Laboratory, Berkeley, California 94720, United States; ⊥ Helmholtz Zentrum München, German Research Center for Environmental Health, 81377 Munich, Germany; # German Center for Infection Research (DZIF), Partner Site Munich, 81377 Munich, Germany; ∇ University of Hamburg, Notkestraße 9-11, 22607 Hamburg, Germany; ○ Department of Physics, University of Hamburg, Notkestraße 9-11, 22607 Hamburg, Germany; ◆ Applied Science Cluster, UPES, 248007 Dehradun, India; ¶ Technology Platform Light Microscopy (TPLM), Universität Hamburg (UHH), 22607 Hamburg, Germany; ⋈ Centre for Structural Systems Biology (CSSB), 22607 Hamburg, Germany; ⧓ Technology Platform Light Microscopy and Image Analysis (TP MIA), Leibniz Institute of Virology (LIV), 20251 Hamburg, Germany; ⧖ Institute of Virology, Technical University of Munich/Helmholtz Munich, 81675 Munich, Germany; ● Department of Otorhinolaryngology, LMU University Hospital, LMU München, 81377 Munich, Germany; ¤ KTH Royal Institute of Technology, Teknikringen 56-58, 10044 Stockholm, Sweden; ☼ The Hamburg Centre for Ultrafast Imaging, Universität Hamburg, Luruper Chaussee 149, 22761 Hamburg, Germany

**Keywords:** SARS-CoV-2 virus-like particles (VLPs), titanium dioxide, photocatalytic oxidation, GISAXS, AFM, XPS

## Abstract

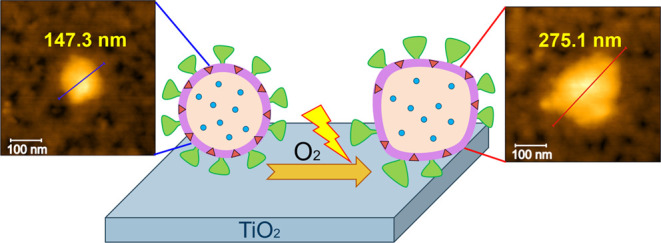

Titanium dioxide (TiO_2_) shows significant potential as a self-cleaning material to inactivate severe acute respiratory syndrome coronavirus 2 (SARS-CoV-2) and prevent virus transmission. This study provides insights into the impact of UV-A light on the photocatalytic inactivation of adsorbed SARS-CoV-2 virus-like particles (VLPs) on a TiO_2_ surface at the molecular and atomic levels. X-ray photoelectron spectroscopy, combined with density functional theory calculations, reveals that spike proteins can adsorb on TiO_2_ predominantly via their amine and amide functional groups in their amino acids blocks. We employ atomic force microscopy and grazing-incidence small-angle X-ray scattering (GISAXS) to investigate the molecular-scale morphological changes during the inactivation of VLPs on TiO_2_ under light irradiation. Notably, *in situ* measurements reveal photoinduced morphological changes of VLPs, resulting in increased particle diameters. These results suggest that the denaturation of structural proteins induced by UV irradiation and oxidation of the virus structure through photocatalytic reactions can take place on the TiO_2_ surface. The *in situ* GISAXS measurements under an N_2_ atmosphere reveal that the virus morphology remains intact under UV light. This provides evidence that the presence of both oxygen and UV light is necessary to initiate photocatalytic reactions on the surface and subsequently inactivate the adsorbed viruses. The chemical insights into the virus inactivation process obtained in this study contribute significantly to the development of solid materials for the inactivation of enveloped viruses.

## Introduction

The COVID-19 pandemic 2019, caused by the severe acute respiratory syndrome coronavirus 2 (SARS-CoV-2), has raised numerous questions for the scientific community.^1^ It has shown that interdisciplinary and multidisciplinary approaches encompassing medicine, material science, technology, biomedical science, chemistry, and applied physics are necessary to comprehend the mechanism behind the worldwide spread of viruses and to control them.^1,2^ One of the potential routes for SARS-CoV-2 transmission is through indirect contact with contaminated surfaces. The virus can remain active on various surfaces for minutes up to hours, depending on environmental conditions.^3,4^ Therefore, effectively controlling the transmission of the virus from surfaces is a key strategy for slowing its dissemination in both indoor and outdoor public environments. Various surface disinfection methods have been reported for virus inactivation, such as alcohol,^5^ heat, and ultraviolet (UV-C) (wavelength: 200–280 nm) irradiation,^6−8^ which inactivate the virus via denaturation, damage, or destruction of the ribonucleic acids (RNA) and proteins.^8^ However, several significant limitations are associated with the aforementioned disinfection methods, concerning their sustainability and safety. For example, the alcohol in antiviral products is not stable over time due to evaporation or dissipation.^9^ Instead, heat (>65 °C) and UV irradiation consume energy and may pose a threat to human health.^8^ Antiviral surfaces are promising technologies with practical applications in public places, such as hospitals, schools, and nursing homes, aimed at preventing the spread and inactivation of transmission viruses without using chemicals or requiring energy.^10,11^ Physical and chemical properties of the surface, such as porosity and hydrophobicity, are the key factors influencing their interaction with viruses. Therefore, there is a high demand to develop and design highly efficient antiviral surfaces using cost-effective and environmentally friendly materials.^12^ Photocatalytic materials represent promising antiviral coatings capable of preventing virus transmission and reducing indoor infections.^13−16^

Titanium dioxide (TiO_2_) stands out as one of the best photocatalytic materials, well-known for its high activity for the degradation of organic compounds, the inactivation of bacteria and viruses, and its self-cleaning properties.^17^ It is nontoxic, commercially available and chemically and thermally stable.^8^ By absorbing light with energy higher than the band gap (∼3.2 eV),^8^ photogenerated electron/hole (e^–^/h^+^) pairs form in TiO_2_ and can react with oxygen and water molecules, producing reactive oxygen species (ROS), such as superoxide (O_2_^•–^) and hydroxyl (HO^•^) radicals.^8,18^ These generated ROS possess high oxidative potential, effectively oxidizing organic components of microorganisms, resulting in cell wall and membrane rupture, RNA destruction, and protein alteration.^19^ Several metal oxide-based photocatalysts, such as CuO^20^ and TiO_2,_^8^ and nanoparticle decorated metal oxides (e.g., Ag/TiO_2_,^21^ Au/ZnO,^22^ and Pd/TiO_2_^8^) have been used for virus inactivation. In most work reported so far, the reaction is performed in the liquid phase in the presence of a catalyst and virus suspension,^9,23,24^ and there is a lack of knowledge concerning the inactivation mechanism.

In our previous study, we demonstrated that the TiO_2_(101) photocatalyst surface can adsorb SARS-CoV-2 and further inactivate it through a combination action of light and thermal treatments.^8^ However, due to biosafety restrictions, we were unable to investigate the adsorption and interaction of the virus with the TiO_2_(101) surface before exposing it to UV light, as we had to inactivate the virus for further characterization outside of the biosafety level 3 (BSL-3) laboratory. Shifting all instrumentation to a BSL-3 laboratory was also unfeasible due to many technical and economic constraints. In order to overcome these limitations, in this work, we focus on the newly developed technology of SARS-CoV-2 virus-like particles (VLPs), which resemble infectious viruses in morphology but lack the viral genome.^25^ VLPs are also promising candidates for virus and vaccine studies (see Figure S1 in the Supporting Information).^25−27^ Therefore, with the present study, we report, for the first time, a combined experimental and theoretical investigation of the adsorption and photocatalytic inactivation of SARS-CoV-2 VLPs on TiO_2_. Furthermore, we reveal the role of light in the denaturation of viral proteins and changes in the membrane morphology of the adsorbed viruses. Such insights into interactions of VLPs with solid material surfaces pave the way for the further development and design of effective antiviral surfaces.

## Results and Discussion

### Adsorption of SARS-CoV-2 Virus-Like Particles (VLPs) on TiO_2_(101) Surface

It was reported in the literature that the SARS-CoV-2 virus can attach to some surfaces through various physical interactions, such as polar, nonpolar, and van der Waals.^8,28^ Moreover, it was suggested that the antiviral properties of a surface are correlated with its ability to adsorb viruses.^12^ As the first step of our investigation, we confirm the interaction and binding of VLPs to the TiO_2_(101) surface by combining fluorescence microscopy (FM), atomic force microscopy (AFM), nanoinfrared (Nano-IR), and X-ray photoelectron (XPS) spectroscopic analysis with density functional theory (DFT) calculations simulating XPS binding energies (BEs). SARS-CoV-2 VLPs, which are sketched in Figure S1, and high-quality TiO_2_(101) surface (single crystal 8 mm × 8 mm × 2 mm, Surface Net Ltd.) have been prepared by repeated sputtering and annealing cycles, as described in our previous study^8^ (see the Supporting Information for more information). In order to confirm the presence of VLPs on the surface, we carried out FM measurements. For this technique, we incubated the samples using rabbit-derived anti-SARS-CoV-2 nucleocapsid primary antibodies and antirabbit secondary antibodies conjugated with the fluorescent-dye Alexa568 (see the Experimental Section for additional details). FM intensity can indicate specific staining arising from the interaction of antibodies with VLPs, while residual nonspecific signals can result from the interaction of antibodies with other proteins adsorbed on the surface.^29^ To prevent antibody binding directly to the surface of TiO_2_, a blocking solution (a mixture of phosphate-buffered saline and bovine serum albumin, BSA) was applied to the sample with the VLPs adsorbed and the respective control samples (see Figure S3, Supporting Information). Mean fluorescence intensities for these measurements were reported in Table S1 (Supporting Information). According to the obtained values, specific staining resulted in a 1.9 times higher fluorescence intensity than the respective controls without primary antibodies or VLPs, confirming the presence of VLPs on the surface of TiO_2_(101).

**Figure 1:**
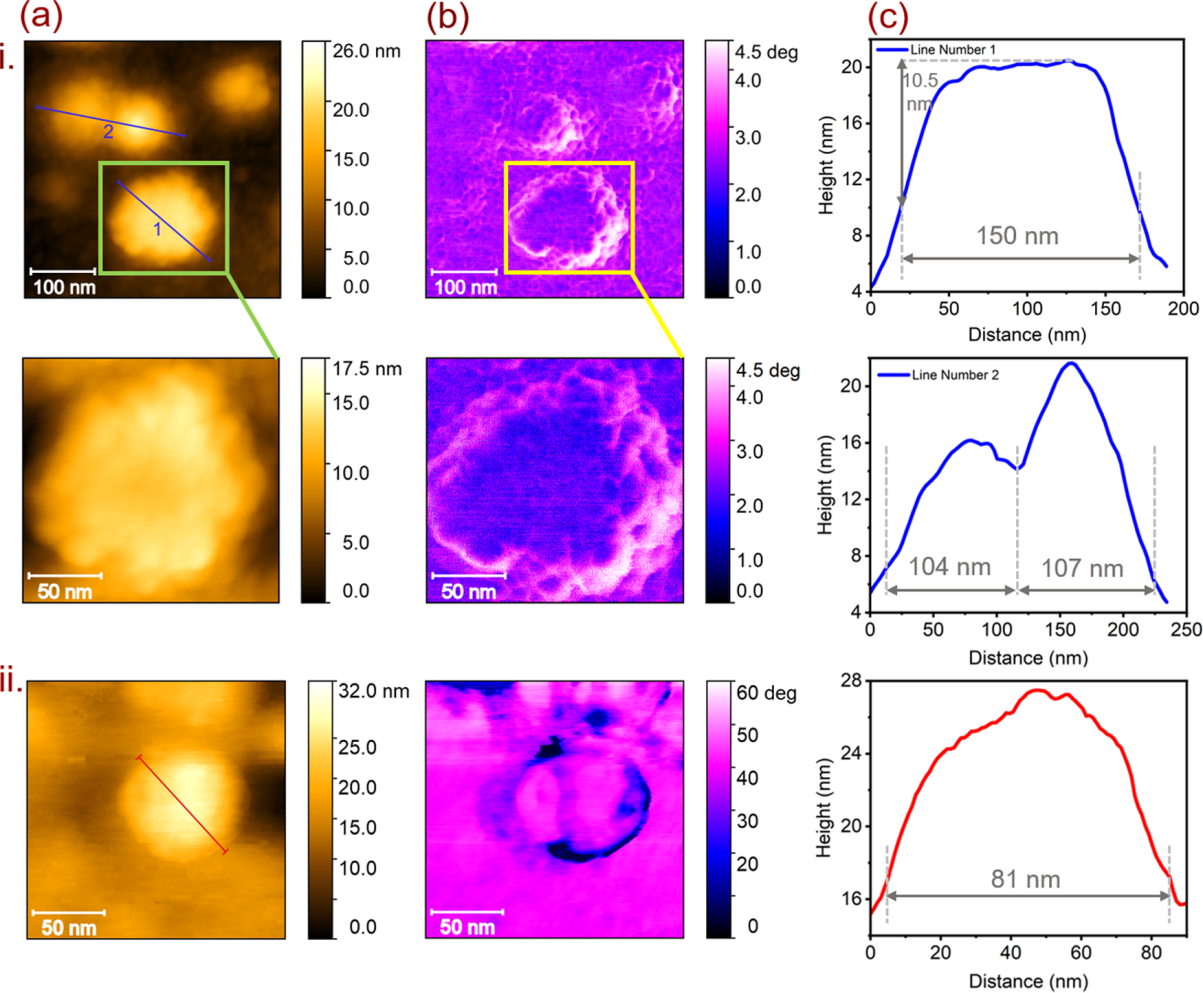
(a) AFM topography (0.4 × 0.4 μm^2^ (i) and 0.15 × 0.15 μm^2^ (ii)) and (b) phase images of adsorbed SARS-CoV-2 VLPs (i) and Δ*S*VLP (ii) on the surface of TiO_2_(101). (c) Line scan profile of selected particles in AFM topography images (a, (i) and (ii)).

To visualize the shape and morphology of the adsorbed VLPs, the sample was characterized using AFM at DESY NanoLab.^30^Figure 1a,b display the AFM and phase images, respectively. The AFM images reveal spherical particles with a particle diameter in the range of 100–150 nm, corresponding to the VLPs (see line scan profiles in Figure 1c). SARS-CoV-2 VLPs have been reported in the size of 60–150 nm by Roessler et al.^25^ The common nonuniform size distribution is due to the engineered nature of VLPs.^25^ Nevertheless, these VLPs exhibit structural and visual similarities to living SARS-CoV-2 viruses,^8^ despite the lack of a complete virus genome. The height of the adsorbed VLPs (Figure 1c) appears to be smaller than the expected diameter (60–150 nm). A similar behavior was observed in the case of adsorbed SARS-CoV-2 on the solid surface of TiO_2_(101).^8^ The height of adsorbed SARS-CoV-2 particles on glass and tissue culture polystyrene substrates was reported in the range of 9–15 and 14–38 nm, respectively.^5^ This height reduction might occur due to two main factors: (i) the drying of virus particles on the surface, causing the VLPs become flatter due to the denaturation of spike and membrane proteins, and (ii) the accumulation of cell culture media (CCM, virus growth media) and other proteins smaller than VLPs on the area surrounding the virus.^5,8^ Additionally, virus proteins often undergo conformational changes upon surface adsorption, resulting in a lower height profile.^31^ This phenomenon is known as the “Vroman effect,” in which the adsorbed proteins are displaced by proteins with higher affinities for the surface.^32^ As proteins interact with a surface, they can bind to the surface. Over time, these adsorbed proteins undergo molecular relaxation or spread on the surface, resulting in enhanced affinity interaction with the surface and consequently shrinking in height.^33^ The spike proteins in SARS-CoV-2 play a crucial role in the virus’s life cycle and its interaction with host cells.^8^ Responsible for mediating viral entry into host cells, the spike protein becomes a key target for antiviral strategies. On average, SARS-CoV-2 is covered by approximately 24–40 spike proteins,^34^ each having a club-like shape measuring approximately 20 nm in length.^35^ The spike protein acts as a major mediator of cellular infection and consists of two distinct subunits, S1 and S2 (see Figure S1, Supporting Information).^8^ The size of the spike protein subunit 1, which contains the receptor binding domain to attach to the angiotensin-converting enzyme 2 receptor on host cell surfaces, has been reported to range between 10 and 15 nm.^31,36^

High-resolution AFM phase images of the VLPs corresponding to the topography images on TiO_2_(101) in Figure 1a are shown in Figure 1b. We observed spiky features along the edges of the virus particles in the phase image, potentially related to spike proteins. To confirm this interpretation, we further characterized engineered subviral particles without spike proteins (Δ*S*VLP) as a reference sample on TiO_2_(101). Figure 1a,b (ii) displays the AFM and phase images of Δ*S*VLPs/TiO_2_(101). The assembled Δ*S*VLP has a spherical shape with a size of 81 nm. Compared to the adsorbed VLPs on the surface of TiO_2_(101), the AFM and phase images of the Δ*S*VLP/TiO_2_(101) sample showed bald particles, providing proof of the presence of spike proteins in VLPs. We can clearly see in the AFM images that the shape and size of VLPs are similar to the SARS-CoV-2.^8,37^

**Figure 2:**
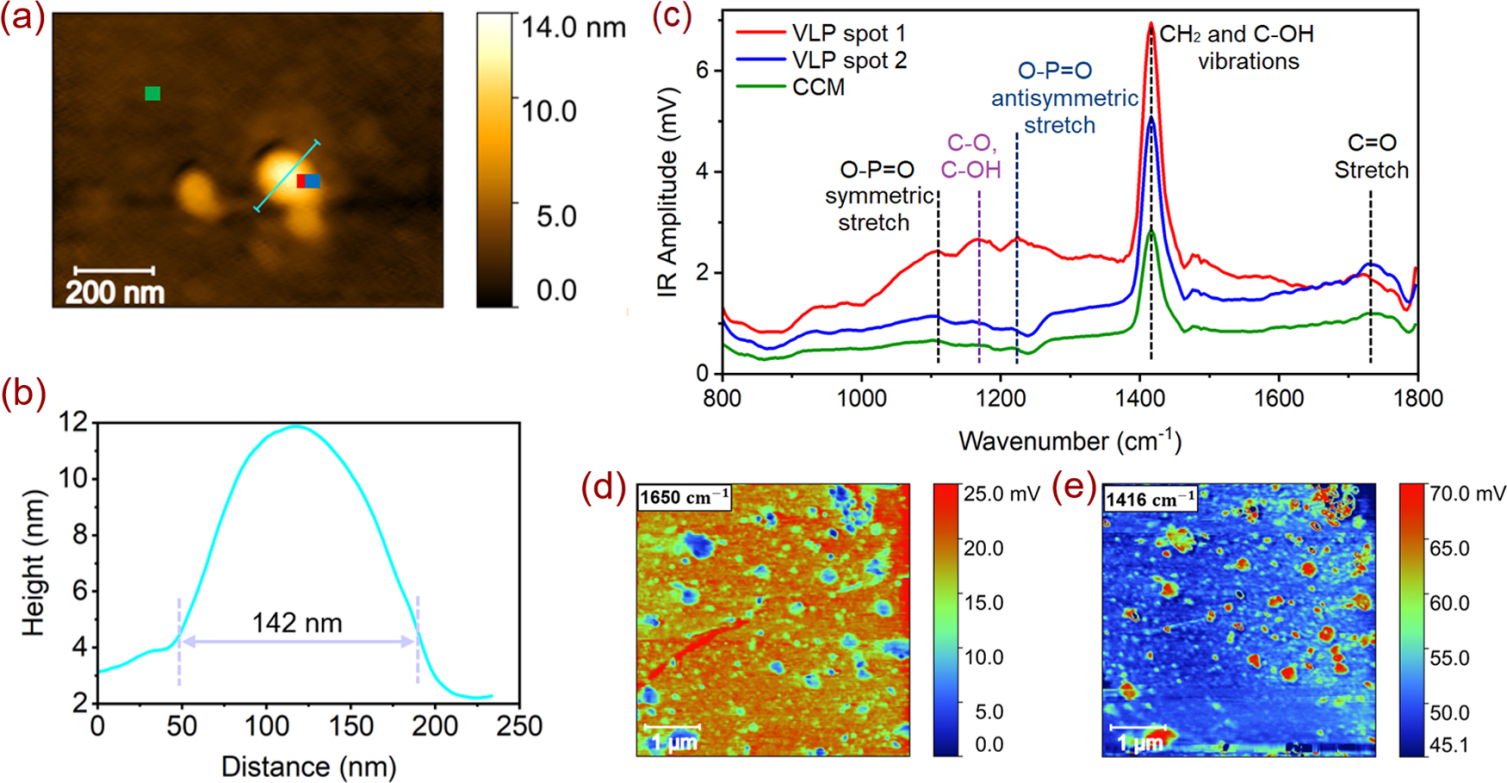
(a) AFM (1.0 × 1.0 μm^2^) topography image of the selected area in Figure S4. (b) Line scan profile of selected VLP for nano-IR measurement. (c) AFM-IR spectra of adsorbed SARS-CoV-2 VLPs on the surface of TiO_2_(101) at three different locations in the AFM image. The red, blue, and green spectra are acquired at positions corresponding to the red, blue, and green spots in the AFM topography image (a). AFM-IR maps were measured at two different frequencies: 1650 cm^–1^ (d) and 1416 cm^–1^ (e).

To obtain more information about the chemical binding of the adsorbed VLPs, AFM-based nanoinfrared (Nano-IR) spectroscopy was employed. Nano-IR enables the combination of AFM and IR techniques for the local visual-chemical composition mapping at the surface.^38^ In the case of the VLPs, the advantage of Nano-IR over bulk IR is the ability to separately identify virus signals from cell culture media (CCM) by the intrinsic spatial resolution, permitting two-dimensional (2D) chemical mapping with nanoscale resolution. In Figure S4, a broad view (5.0 × 5.0 μm^2^) of the surface displays many VLPs and a few self-assembled nanocrystals formed from the buffer medium during the sample drying after loading the VLPs. The AFM topography of a single virus particle and its measured line scan profile is shown in Figure 2a,b. The size of the selected VLP in this measurement is 142 nm in length and 8 nm in height. The spectra in Figure 2c were taken at two different locations inside the VLP (see the red and blue spots in the AFM image). To distinguish the information about the chemical binding of VLPs from that of the CCM, we also characterized a spot at the surface that is VLP-free as a reference containing only CCM (see the green spot in the AFM image). The strong band at 1416 cm^–1^ in the VLP spectrum, which exhibits lower intensity in the CCM spectrum, can be assigned to the CH_2_^39^ and C–OH^40^ vibrations originating from the protein amino acids structure within the viral particle. Since the CCM also contains amino acids and vitamins,^8^ this vibration band was also observed at VLP-free spots as well. The IR band centered around 1734 cm^–1^ in the spectra is associated with the C=O stretching vibrations.^41^ The main parts of the virus such as lipids, RNA, and carbohydrates exhibit adsorption bands at 1290–1050 cm^–1^ region in the IR spectra.^41,42^ Contrary to the CCM spectrum, the observed broad bands at 1232, 1168, and 1105 cm^–1^ are attributed to the O–P=O antisymmetric stretching vibration,^41^ the functional groups of proteins (C–O stretching mode of the C–OH group),^43^ and O–P=O symmetric stretching vibration and C–N,^41,44,45^ respectively (Figure 2c). The main source of the P–O vibration is the nucleocapside protein in the VLP structure. AFM-IR nanoimaging was then carried out at two different IR frequencies: at the amide peak associated with protein at 1650 cm^–1^ (Figure 2d, protein map) and at the vibration of the CH_2_ groups at 1416 cm^–1^ (Figure 2e, CH_2_ map). The protein cargo of an individual virus particle was identified by local nano-IR measurement on the surface of solid TiO_2_. The nano-IR results confirm the presence of viral proteins by observing the different bands of functional groups in lipids and proteins of VLPs, compared to those in CCM.

**Figure 3:**
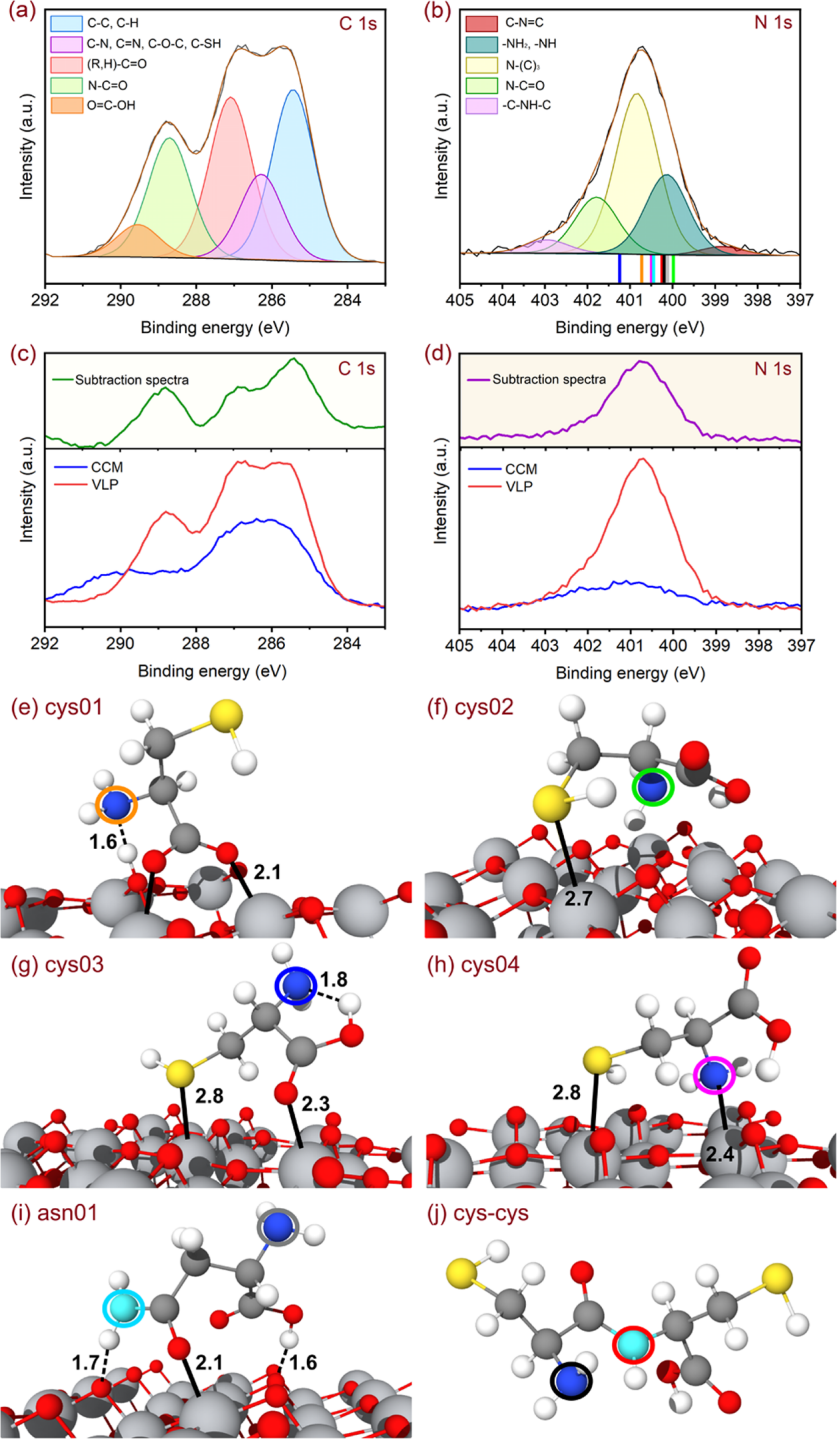
C 1s (a) and N 1s (b) spectra of adsorbed CCM and VLPs on the surface of TiO_2_(101) and on top difference spectra of VLPs and CCM are included for the C 1s and N 1s core-level scans. Deconvoluted core-level photoelectron spectra of C 1s (c) and N 1s (d) for adsorbed VLPs on the surface of TiO_2_(101). In panel (b) the position of the peaks calculated for the different N species and adsorption modes is marked by colored sticks. The structure of the optimized DFT models of the simulated adsorbed cys or asn molecules as well as that of the isolated dipeptide are shown in panels (e–j). N, O, C, S, Ti, and H atoms are shown by blue, red, gray, yellow, (larger) gray, and white spheres, respectively. Each N atom is highlighted through circles, whose colors correspond to that of the sticks in panel (b). On all of the structures, chemical or hydrogen bonds are highlighted by solid or dashed black lines, respectively. The corresponding bond length is reported as well in Å.

It was reported that the binding and interaction of SARS-CoV-2 with nonbiological surfaces are correlated with the virus stability and its potential for surface transmission in indoor environments.^46^ Therefore, gaining a deeper understanding of the interaction between various components of the functional groups in SARS-CoV-2 proteins and surfaces is crucial for developing effective strategies to mitigate virus transmission and functionalized surfaces. In addition to Nano-IR, the adsorbed VLPs on the surface of TiO_2_(101) are characterized using both experimental and simulated XPS (see characterization techniques section). Figure 3a,b reports high-resolution XP spectra of the C 1s and N 1s core levels. The deconvoluted XP spectrum of C 1s, which shows the contribution of lipid and proteins, is fitted with five components (Figure 3a) at binding energies (BEs) centered at 285.4 eV (C–C and C–H bonds^8^), 286.3 eV (C–N and C=N,^8,47^ C–O–C,^48^ and C–SH^8^), 287.1 eV ((R, H)–C=O bond^48^), 288.7 eV (N–C=O^49,50^), and 289.5 eV (O=C–OH^8^). These species are present in different amino acids and proteins within the structure of VLPs, such as spike proteins, nucleocapsid proteins, and lipid envelopes. However, since the VLPs sample contains CCM, the adsorbed CCM on the surface of TiO_2_(101) was separately characterized using XPS to distinguish the contributions from the VLPs and those from the CCM. Figure 3c,d shows the C 1s and N 1s core-level spectra for the VLPs and CCM samples as well as the corresponding difference spectra. These results can be compared with those we reported in our previous study on the adsorption of the SARS-CoV-2 virus on the surface of anatase TiO_2_(101).^8^ In the case of the core levels of C atoms, the spectrum of the VLPs agrees with that measured for the inactivated virus, with slightly different BEs 285.4 vs 284.5, 287.1 vs 286.5 eV and 289.5 vs 287.9 eV but similar energy separation 1.7 vs 1.6 eV and 1.4 vs 2.4 eV. The minor discrepancies in the intensity and the position of the peaks can be ascribed to a different folding of the proteins since in the case of inactivated virus, they were partly denatured under the thermal treatment. In our previous study, we concluded, based on the results of the DFT calculation, that the XP C 1s core level carries the fingerprints of the SARS-CoV-2 adsorbed on TiO_2_(101). Therefore, we assume these results to be valid for the VLP, which has the same shell structure as SARS-CoV-2. In the case of the N 1s contributions, the deconvolution of the XP spectrum reveals five components, centered at 398.8, 400.1, 400.8, 401.8, and 402.9 eV, characteristic of C–N=C,^51^ –NH_2_/–NH,^51^ N-(C)_3_,^51^ N–C=O,^52^ and –C–N–C–,^8^ respectively. After the removal of the contribution of the CCM, among the features related to the VLPs only (Figure 3d at 400.1, 400.8, and 401.8 eV), the first and the last peaks have an energy separation similar to that measured for the inactivated virus (1.7 vs 1.9 eV). On the contrary, the intermediate peak is observed exclusively for the VLP, with a BE 0.7 eV higher than the feature with the lowest BE. We model the adsorbed VLP with two of the most abundant amino acids in the spike proteins, cysteine (cys), and asparagine (asn), similarly to what some of the authors did for the inactivated virus.^8^ These model structures are shown in Figure 3e–i. In addition, we calculated the XPS signature of a cys–cys dipeptide, and its optimized structure is displayed in Figure 3j, in order to include the prototypical contribution of the secondary amide group involved in the peptide bonds forming the amino-acid chains in the spike protein. In this case, we did not investigate any interaction between such a functional group and the surface, since we assumed that the N amide was not exposed to the environment by the folded protein. The positions of the simulated XPS features are reported in Figure 3b through colored sticks. Our numerical approach only allows us to calculate the BE differences or core-level shift (CLS); therefore, we have aligned the lowest contributions measured experimentally with the fit component with the lowest BE calculated by DFT, while the numerical values of the CLS are collected in Table S2. Additional information about the CLS calculation can be found in the Supporting Information.

The analysis of the correlation between the CLS of the different N atoms and their interactions with the surroundings allows us to effectively separate the calculated XP peak contributions into three groups, which qualitatively agree with those peaks deconvoluted from the experimental spectrum. In the first group, which we assign to the feature centered at 400.1 eV, we include the secondary amide group of the peptide bonds (as in the cys–cys model) and the amino groups with no interaction with the surface or other groups of the molecule itself (as in the cys02 and asn01 models). This attribution is supported by the fact that these CLSs are in agreement with the amino group of the isolated cys–cys dipeptide. In the second group, which we assign to the feature at the highest BE at 401.8 eV, we include the N groups, both amino and amide, close to a carbonyl group chemically bonded to a surface Ti atom (as in the cys03 model). Finally, we define a last group at intermediate BE, which we assign to the feature centered at 400.8 eV, where we include the amino and the amide groups that interact directly with the surface or other groups and also are located close to a carbonyl group bound to the surface as in cys01, cys04, and asn01 models. These atoms would experience two opposite effects, namely an increase of BE, triggered by the adsorbed carbonyl group nearby, and a red shift in BE, due to the formation of H-bonds as previously was observed in H-bonded amino groups in carbon nitride polymers.^53^ Therefore, these results support the adsorption of VLPs with spike proteins and that our synthesized VLPs can be considered reliable for studying the adsorption of the SARS-CoV-2 virus on the surface of TiO_2_(101).

### UV-Induced Transformation of SARS-CoV-2 VLPs/TiO_2_(101) System

**Figure 4:**
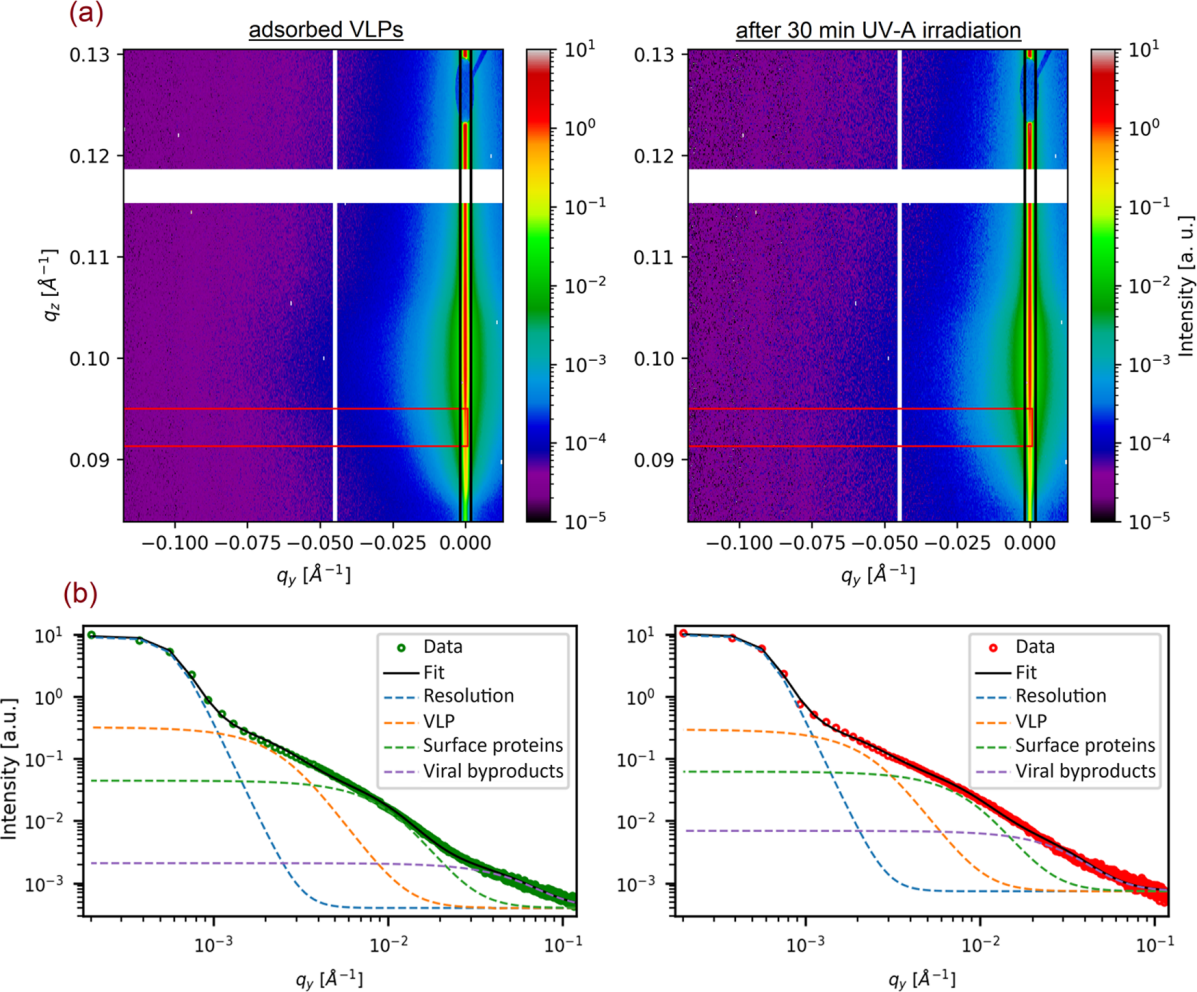
(a) 2D GISAXS data of the adsorbed VLPs on the surface of TiO_2_(101) and after 30 min of UV irradiation in air. The black and red boxes mark the vertical and horizontal cut position, respectively. (b) Horizontal line cuts at the region of interest (red box in (a)) with the corresponding fit.

Recently, we discovered that UV-C (wavelength: 265 nm) can effectively inactivate SARS-CoV-2 adsorbed on a TiO_2_(101) surface through viral genome damage and photocatalytic oxidation.^8^ Since we characterized the sample after applying the inactivation method, we were unable to distinguish the photocatalytic inactivation mechanisms from the denaturation processes. Understanding the role of surface chemistry in virus inactivation is crucial, especially for the development of efficient self-cleaning materials for surface cleaning. To understand the role of TiO_2_(101) surface in the inactivation of the virus under UV light, we performed AFM and grazing-incidence small-angle X-ray scattering (GISAXS) experiments before and after UV treatment. These microscopic and scattering techniques provide us with more insights into the effect of light on the virus at the oxide interface, helping to understand the possible catalytic inactivation mechanisms of the virus. The GISAXS experiments were performed at beamline P03 of the synchrotron facility PETRA III (DESY, Hamburg) (see characterization techniques section).^54,55^ After loading the VLPs onto the surface of TiO_2_(101) (single crystal 8 mm × 8 mm × 2 mm), a GISAXS pattern was collected at room temperature in the air before exposing the sample to UV-A light (wavelength: 365 nm, intensity: 850 μW/cm^2^) and again after 30 min of UV exposure. To reduce the effects of temperature and light damage during sample transfer from the biosafety level 2 (BSL-2) laboratory to the beamline, the samples were transported in a closed dry ice bath to avoid viral protein denaturation. For studying the interaction of X-rays with the VLPs, one spot on the sample surface was continuously illuminated by the high-flux X-ray beam for 20 s (see Figure S7 in Supporting Information). During this time, a detector image (Figure 4a) was taken every 0.1 s. From each image, a horizontal (red box in Figure 4a) and vertical cut (black box in Figure 4a) was extracted. The position and width of the horizontal and vertical cuts were chosen to visualize X-ray-induced changes in the VLP morphology and sample composition, respectively. It is evident that continuous illumination for 20 s (see Figure S7), results in no X-ray-induced changes.

In order to not limit the study of the VLP morphology to one spot (perpendicular × parallel to the beam direction: 0.03 × 5 mm^2^) but over the whole sample surface, a total of 50 scattering images (illumination time: 0.1 s) were collected with a spacing of 100 μm between them over the whole VLP-loaded surfaces before and after the UV treatment. Consequently, all images were summed to provide averaged information regarding the VLP morphology across the whole sample surfaces. Since the main focus was on the in-plane structural changes of the VLP, only the horizontal cuts (red box in Figure 4a) were analyzed. Here, the proteins and VLPs sizes and size distribution were determined using the so-called effective interface approximation of the distorted wave Born approximation.^56−58^ For modeling, a combination of a Lorentzian resolution function (centered at *q*_*y*_ = 0 Å^–1^) and three particle populations were assumed, which were described using spherical form factors with a Gaussian size distribution known from general small-angle X-ray scattering theory.^59^

**Figure 5:**
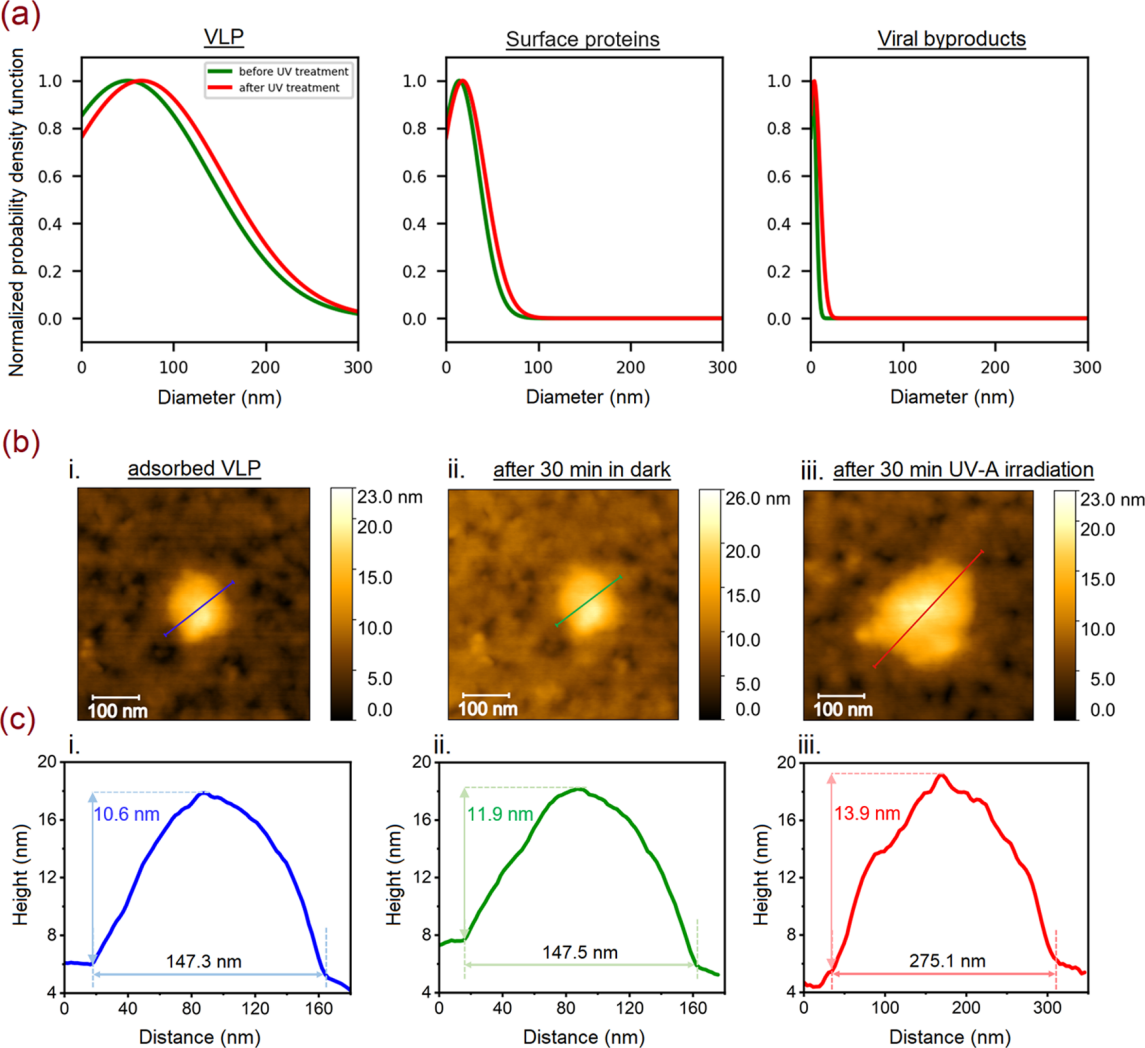
(a) Particle size distribution of VLP, surface proteins, and viral byproducts obtained from fitted GISAXS scattering curves in Figure 4b. (b) AFM topography images (0.5 × 0.5 μm^2^) of a single selected adsorbed VLP on the surface of TiO_2_(101) (i), after 30 min in dark and air (ii), and after UV irradiation in air for 30 min (iii). (c) Line scan profile of the particles in the corresponding AFM topography images in (b).

Considering the denaturation of spike proteins and other viral proteins in the virus structure during the adsorption process on the surface of TiO_2_(101),^8^ we examined three different models to fit data to cover all particles adsorbed on the surface, (i) VLPs, (ii) surface proteins (the spike, membrane, and envelope proteins), and (iii) viral byproducts. In contrast to the clean surface of TiO_2_(101), the intensity of the GISAXS curve for adsorbed VLPs is high, confirming the adsorption of VLPs (see Figure S9 in the Supporting Information). Based on the GISAXS curves, the intensity changed after UV treatment. Since the bulk beam does not affect the virus morphology (according to the beam damage scan results), the change in intensity is attributed to the effect of UV irradiation on the virus structure. By fitting the GISAXS curve (Figure 4b), we determined the average diameters of VLPs, surface proteins, and viral byproducts as 50.2, 14.4, and 2.5 nm, respectively (Figure 5a, for more information, see Table S3). After exposing the system to light, we observed an increase in the average diameter of both VLPs and surface proteins, suggesting that UV light has an effect on the size of the adsorbed particles. To delve deeper into the effect of UV irradiation, we performed a similar experiment using AFM and measured one adsorbed VLP before and after exposing the sample to light (Figure 5b). After VLPs were loaded onto the surface of TiO_2_(101), one adsorbed virus particle was measured with AFM, which exhibited a spherical particle with a size of 147.5 nm (see the line scan in Figure 5c). A control experiment was carried out by keeping this sample exposed to air and in dark conditions for 30 min, followed by characterizing the same particle using AFM. During the control experiment, the target particle exhibited no changes in shape and size and stayed intact (Figure 5b,c-ii). However, after 30 min of exposure to UV-A (Wavelength: 365 nm), the diameter of the particles increased and its spherical structure disappeared (Figure 5b,c-iii). The UV-A exposed VLP/TiO_2_(101) sample showed a particle with a size of 275.1 nm. Based on the line scan profile, the observed island on the line depicted in Figure 5c-iii exhibits a size ranging from 15 to 25 nm. This range of particle size may correspond to the surface proteins that dissociated from the virus during the photocatalytic reaction and/or as a result of the viral protein denaturation process at the surface of the oxide photocatalyst. According to the AFM results, the height of the selected particle decreased from 11.9 to 10.6 nm after the sample was kept in the dark due to the Vroman effect. However, after exposing the particle to UV-A light for 30 min, its height increased and reached 13.9 nm. This increase could be related to the polymerization of the dissociated surface proteins or membrane rupture during UV irradiation. The *in situ* AFM results, in line with the GISAXS findings, elucidate the photoinduced changes in the VLPs morphology on the TiO_2_(101) surface. When we conducted experiments both in dark conditions and under UV irradiation in air, no changes were observed in VLP morphology without UV light. This suggests that changes in VLP morphology occur only under oxygen pressure (air) combined with UV irradiation. There are two reactions that can take place on the surface of TiO_2_/VLPs sample under light and air exposure: (i) the denaturation of structural proteins induced by UV irradiation, leading to readsorption and cross-linking interactions between amino acids and (ii) the oxidation of the virus structure through photocatalytic reactions by producing ROS in the interaction of air/VLP/TiO_2_. In both cases, TiO_2_ acts as a photocatalyst providing an active surface for the photoinduced changes in VLPs. Protein denaturation refers to the alteration of a protein’s native structure, leading to the loss of its functional conformation. This can manifest as changes in size, including unfolding or aggregation of the protein. Additionally, the presence of ROS at the surface of the catalyst can induce modifications in the morphology of microorganisms.^19^ ROS have been reported to induce oxidative damage in viruses and bacteria, affecting various components such as proteins, RNA, DNA, and lipid membranes.^60^ ROS can impact the lipids, RNA, and cell membrane by selectively attacking nucleotides and sulfhydryl bonds. The result will be the breakdown of the cell wall, which subsequently leads to changes in the size and shape of the virus structure. For instance, it has been reported that the interaction between HO^•^ radicals and *Bacillus* spore cells can disrupt their structure, changing their spherical shape and causing cell death.^19^ Additionally, the photogenerated holes in the valence band of TiO_2_ exhibit potent oxidizing properties capable of oxidizing the surface proteins, subsequently deactivating them.^61^ Air humidity can significantly affect the generation of hydroxyl radicals. In the presence of humidity, water molecules adsorb onto the surface of TiO_2_ and increase the surface hydroxyl groups after dissociation.^62^ These hydroxyl groups can then be transformed into radicals under UV irradiation, which enhances the photocatalytic reaction. The air humidity during the measurements was 65% in Hamburg, Germany.

To confirm that the generated ROS are responsible for morphological changes through oxidation reactions, the effect of UV-A light on adsorbed VLPs was studied using the GISAXS method under an N_2_ atmosphere. After measuring the adsorbed VLPs in a N_2_ atmosphere, the sample was exposed to UV-A light while still under an N_2_ atmosphere to observe the effects of UV-A without oxygen on VLP morphology. GISAXS curves demonstrated no changes in the structure of VLPs under UV-A in an N_2_ atmosphere (see Figure S9 in the Supporting Information). This provides evidence that UV-A in the absence of oxygen cannot affect the morphology of adsorbed VLP on the surface of TiO_2_. Therefore, the presence of both light and oxygen is necessary to activate the surface of TiO_2_ and initiate photocatalytic oxidation. Our results suggest that oxygen and consequently the generated ROS on the surface of TiO_2_(101) play a critical role in inducing morphological changes and subsequently inactivating the virus through membrane oxidation.

## Conclusions

We demonstrated the adsorption of SARS-CoV-2 VLPs on the surface of TiO_2_ as a photo active catalytic surface. XPS analysis revealed that the virus can interact with the surface by binding between amine (–NH_2_) and amide (–O=C–NH–) groups in the protein block structure, which was also confirmed by theoretical DFT simulations. We then used the TiO_2_(101)/VLP system under ambient conditions to observe the effects of UV-A light on VLP morphology. Our *in situ* GISAXS and AFM measurements confirmed the photoinduced changes in the morphology of the adsorbed VLPs. This is primarily due to the generation of ROS, which we propose contributes to the oxidation of the envelope protein in the virus structure. Our study elucidates the role of oxygen and subsequent ROS generation in inducing morphological changes in adsorbed VLPs at the surface of TiO_2_, leading to the effective inactivation of the virus. To the best of our knowledge, this study represents the first report providing visual confirmation of the structural damage and morphological alterations of SARS-CoV-2 VLPs at the surface of a solid photocatalyst, using AFM and GISAXS. These results emphasize the potential of such approaches in developing strategies for mitigating viral transmission in various environments.

## Experimental Section

### Preparation of SARS-CoV-2 Virus-Like Particles (VLPs)

To generate *S*^+^ VLPs containing appropriate levels of SARS-CoV-2 B.1 spike (D614G) and SVLPs controls, HEK293T cells were transfected using TransIT-293 (MIR2700, Mirus) according to the manufacturer’s protocol with carefully adjusted ratios of codon-optimized expression vectors coding for spike (S), membrane (M), nucleocapsid (N), and envelope (E) proteins. The expression plasmids are termed p7413.1 (S:D614G), p5025 (mock), p7395.LA3 (M), p7396.NA9 (E), and p7391.MA5 (N), respectively (Figure S1, see the Supporting Information). Media exchange was performed 6 h after the addition of the transfection mix to reduce a potential carryover of plasmid DNA. After 72 h, S^+^ VLPs, S VLPs, or control EVs were harvested from cell culture supernatants (DMEM, 8% FBS, supplemented with Pen/Strep, Gibco, Thermo Fisher Scientific) after low-speed centrifugation at 7 °C for 10 min at 300*g* and 20 min at 4200*g* and generally used without further processing. Particle preparations were flash frozen in liquid nitrogen for storage at −80 °C and subsequently characterized as described by Roessler et al.^25^

### Characterization Techniques

#### Fluorescence Microscopy (FM)

To understand the specific and nonspecific binding of VLPs on the surface of TiO_2_, the sample was characterized by FM. In order to reduce nonspecific antibody binding, the sample including SARS-CoV-2 VLPs was covered with blocking solution phosphate-buffered saline (PBS) supplemented with saponin (0.25%) and bovine serum albumin (BSA, 3.0%). After 30 min, the sample was washed three times with PBS. Then, 20 μL of blocking solution containing 1:200 diluted primary antibody (anti-SARS-CoV-2 nucleocapsid antibody produced in rabbit, Thermo Fisher Scientific) was added to the sample. After incubation time (60 min), the sample was washed with PBS to remove unbound antibody, and then, blocking solution containing 1:500 fluorescent-dye conjugated secondary antibody (antirabbit antibody conjugated with Alexa568, Thermo Fisher Scientific) was added. After incubation (30 min) and washing (three times with PBS), the samples were examined with a fluorescence microscope (Leica DMi8 equipped with a 20× NA 0.4 air objective, a Leica DFC9000 GT sCMOS camera, and a Lumencore Sola SE FISH 365 LED light source). Figure S3 shows the sample preparation process for FM measurements (see the Supporting Information file).

#### Atomic Force Microscopy (AFM)

The morphology of adsorbed SARS-CoV-2 VLPs on the surface of TiO_2_(101) was investigated by AFM at DESY NanoLab.^30^ The AFM and phase images were recorded with different scan sizes (1.5 × 1.5 μm^2^ and 0.5 × 0.5 μm^2^) and 0.996 Hz rate under tapping mode. A RTESPA-300 silicon cantilever from Bruker with a nominal tip radius of 8 nm was used for the measurements. The visualization of measurements were done by the Gwyddion software package.^63^

#### AFM-Based Nanoinfrared (Nano-IR) Spectroscopy

Nano-IR spectroscopy (Anasys Instruments-Bruker) was used to probe the structural composition of adsorbed VLPs on the surface of TiO_2_(101) with nanoscale resolution. AFM-IR spectra were obtained with gold-coated dielectric probes (nominal tip radius: 20 nm) in tapping mode at a scan rate of 0–5 Hz. Analysis Studio and Gwyddion software^63^ were used to export the IR spectra and AFM image, respectively.

#### X-Ray Photoelectron Spectroscopy (XPS)

The XPS measurements of adsorbed VLPs on the surface of TiO_2_(101) was carried out using the XPS system at the DESY Nanolab, at the Centre for X-ray and Nano Science.^30^ The X-ray source employed was Al K_α_ at 1486.6 eV and a Phoibos 150 hemispherical energy analyzer with a base pressure of 1.2 × 10^–10^ mbar.

#### Computational Methods

We performed all calculations within the density functional theory (DFT) framework, using the QuantumESPRESSO suite.^64,65^ We modeled the interaction of a SARS-CoV-2 spike protein with the TiO_2_ anatase (101) considering different adsorption configurations of two of its most abundant amino acids, i.e., cysteine (cys) and asparagine (asn), similarly to those included in a previous work of some of the authors.^8^ In addition, we included in this work the model of an isolated prototypical molecule including the amide bond, i.e., the cys–cys dimer. A sketch of the three isolated molecules is shown in Figure S6 (see the Supporting Information file). For the ground state of all our models, we employed a plane-waves basis set with a cutoff of 52 and 575 Ry for the wave functions and the charge density, respectively, and the PBE exchange-correlation functional. We included the dispersion interactions through the Grimme-D3 pairwise correction.^66^ We modeled the TiO_2_ anatase (101) substrate as a slab including three triatomic layers, with lattice parameters fixed to those of the optimized bulk unit cell (*a* = 3.790 Å and *b* = 10.325 Å). We separated one slab from its periodic replicas along the z direction perpendicular to the surface by inserting a vacuum region 11 Å thick, and we optimized the two topmost triatomic layers, keeping the lowest layer of atoms fixed. The reciprocal space was sampled through a shifted Monkhorst–Pack 2 × 2 × 1 *k*-points mesh.

The simulation of the XPS features at the N K edge was carried out by calculating the core-level shifts (CLSs) using the Δ*S*CF method.^67^ We employed an additional N pseudopotential including a full core hole at its 1s state, requiring an extended basis set cutoff of 74 Ry for the wave functions. Since absolute values of the binding energy are forbidden with this methodology but only relative energy differences (the CLSs), we provided a common energy reference for all the models by including a N_2_ molecule far from the surface. Therefore, all supercells were enlarged along one (in the case of the adsorbed molecules) or more (in the case of isolated molecules) lattice directions in order to obtain a separation from the added reference molecule of at least 15 Å. Such an approach already gave satisfactory results in previous works.^68,69^ The CLS calculated for the different N species in our models are reported in Table S2.

#### Grazing-Incidence Small-Angle X-Ray Scattering (GISAXS)

For the GISAXS measurements, the X-ray wavelength was fixed at 1.05 Å (*E* = 11.8 keV) and the X-ray beam was focused to a 25 × 30 μm^2^ (vertical × horizontal direction) spot. The scattered photons were collected by using a Pilatus 2 M detector (DECTRIS, Baden, Switzerland), which was positioned roughly 5.5 m away from the samples. The incident angle was fixed at 0.4 deg for all measurements.

## Supporting Information Available

Schematic structure of SARS-CoV-2 and SARS-CoV-2 virus-like particles (VLPs). LEED and XP spectra of the clean anatase TiO_2_(101) surface; sample preparation process for fluorescence microscopy (FM) measurements and FM signal for different experiments; AFM topographic image of a selected area on the surface for nano-IR measurement; deconvoluted O 1s and Ti 2p core-level XP spectrum of adsorbed VLPs on TiO_2_(101) surface showing the presence of different functional groups of virus proteins, which interact with the surface of the catalyst. Sketch of isolated cysteine and asparagine monopeptides and cysteine dimer with corresponding calculated core-level shifts (CLS) for different N species in these models; beam damage scan showing the bulk beam cannot affect on virus morphology during the GISAXS measurements; GISAXS detector image showing the good sample alignment at the beamline during the GISAXS measurements; GISAXS data fitting parameters for adsorbed VLPs on TiO_2_ surface before and after UV-A irradiation are reported in Table S3; horizontal line cuts at the region of interest in 2D GISAXS for the adsorbed VLPs and after 30 min UV-A irradiation under air and N_2_ showing that the presence of O_2_ and UV light are necessary to make the surface of TiO_2_ active (PDF)

## Abbreviations

SARS-CoV-2: severe acute respiratory syndrome coronavirus 2
UV: ultraviolet
RNA: ribonucleic acid
TiO_2_: titanium dioxide
ROS: reactive oxygen species
O_2_^•–^: superoxide radical
HO^•^: hydroxyl radical
BSL-3: biosafety level 3
VLPs: SARS-CoV-2 virus-like particles
FM: fluorescence microscopy
AFM: atomic force microscopy
XPS: X-ray photoelectron spectroscopy
UHV: ultra high vacuum
CCM: cell culture media
DFT: density functional theory
cys: cysteine
asn: asparagine
CLS: core-level shift
GISAXS: grazing-incidence small-angle X-ray scattering
