# Supplementary material for: Light-Induced Transformation of Virus-Like Particles on TiO2
Source: ACS Appl Mater Interfaces. 2024 Jul 3;16(28):37275–87. doi: 10.1021/acsami.4c07151 (PMC11261565; doi:10.1021/acsami.4c07151)
Supplement: Supplementary file 1 — am4c07151_si_001.pdf [file am4c07151_si_001.pdf]

# Supporting Information: Light-Induced Transformation of Virus-Like Particles on $TiO_2$

Mona Kohantorabi,[*,†] Aldo Ugolotti,[‡] Benedikt Sochor,[¶,§] Johannes Roessler,[‖,⊥] Michael Wagstaffe,[†] Alexander Meinhardt,[†,#] E. Erik Beck,[†,#] Daniel Silvan Dolling,[†,#] Miguel Blanco Garcia,[†,#] Marcus Creutzburg,[†] Thomas F. Keller,[†,@] Matthias Schwartzkopf,[¶] Sarathlal Koyiloth Vayalil,[¶,△] Roland Thuenauer,[∇,††,‡‡] Gabriela Guédez,[††] Christian Löw,[††] Gregor Ebert,[¶¶] Ulrike Protzer,[¶¶] Wolfgang Hammerschmidt,[‖,⊥] Reinhard Zeidler,[‖,§§,⊥] Stephan V. Roth,[¶,‖‖] Cristiana Di Valentin,[‡] Andreas Stierle,[†,@] and Heshmat Noei[*,†,⊥⊥]

*†Centre for X-ray and Nano Science CXNS, Deutsches Elektronen-Synchrotron DESY, 22607 Hamburg, Germany*
*‡Dipartimento di Scienza dei Materiali, Università degli Studi di Milano-Bicocca, Via Cozzi 55, Milano 20125, Italy*
*¶Deutsches Elektronen-Synchrotron DESY, Notkestr. 85, 22607 Hamburg,*
1 *Germany*
*§Advanced Light Source, Lawrence Berkeley National Laboratory, Berkeley, 94720, CA, USA*
*‖Helmholtz Zentrum München, German Research Center for Environmental Health, 81377 Munich, Germany*
*⊥German Center for Infection Research (DZIF), Partner Site Munich, 81377 Munich, Germany*
*#University of Hamburg, Notkestraße 9-11, Hamburg, 22607, Germany*
*@Department of Physics, University of Hamburg, Notkestraße 9-11, 22607 Hamburg, Germany*
*△Applied Science Cluster, UPES, Dehradun, 248007, India*
*∇Technology Platform Light Microscopy (TPLM), Universität Hamburg (UHH), Hamburg, 22607, Germany*
*††Centre for Structural Systems Biology (CSSB), Hamburg, 22607, Germany*
*‡‡Technology Platform Light Microscopy and Image Analysis (TP MIA), Leibniz Institute of Virology (LIV), Hamburg, 20251, Germany*
*¶¶Institute of Virology, Technical University of Munich/Helmholtz Munich, Munich 81675, Germany*
*§§Department of Otorhinolaryngology, LMU University Hospital, LMU München, 81377 Munich, Germany*
*‖‖KTH Royal Institute of Technology, Teknikringen 56-58, 10044 Stockholm, Sweden*
*⊥⊥The Hamburg Centre for Ultrafast Imaging, Universität Hamburg, Luruper Chaussee 149, 22761 Hamburg, Germany*

E-mail: mona.kohantorabi@desy.de; heshmat.noei@desy.de

## Preparation of SARS-CoV-2 VLPs

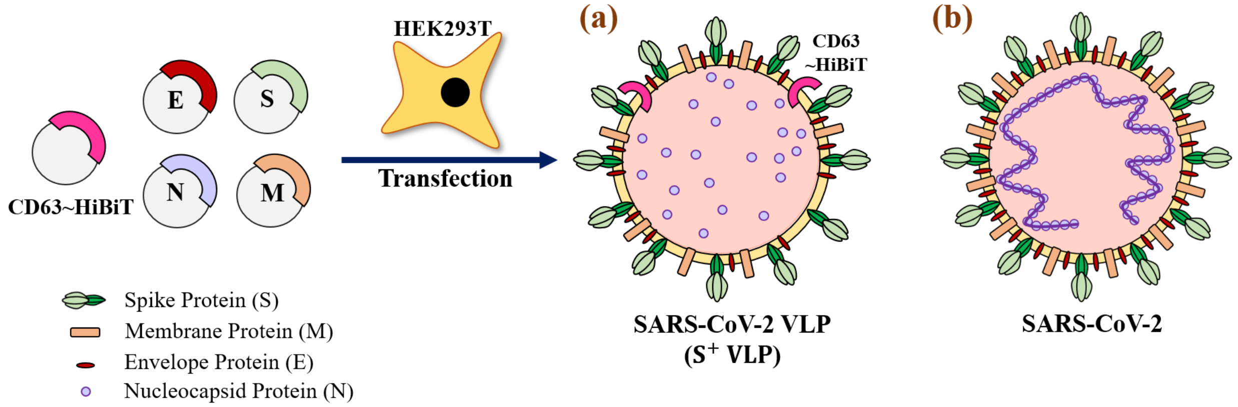


Figure S1: (a) Schematic structure of SARS-CoV-2 virus like particle (VLP) was generated by transfection of HEK293T cell with an optimized ratio of structural proteins S, M, N, and E in the presence of activator peptide (CD63 HiBiT). (b) Schematic view of SARS-CoV-2 virion.

# Preparation of $TiO_2(101)$

The $TiO_2(101)$ single crystal (8 mm × 8 mm × 2 mm, Surface Net Ltd.) was prepared by 1.0 kV $Ar^+$ ion bombarated and 850 K anneal cycles in a backpack pressure of 1 $\times 10^{-6}$ mbar of $O_2$. This cleaning cycle was repeated until a sharp (1×1) low energy electron diffraction (LEED) pattern was obtained and XPS showed a clean surface free of contamination without any $Ti^{3+}$ defects (Figure S2 a, b). These measurements were carried out using XPS setup in the DESY Nanolab at the Centre for X-ray and Nano Science (CXNS).[1] After the experiment, the surface was cleaned in low concentrated acid solution and then we repeated the sputtering and annealing cycles. The sample was characterized by XPS. According to the XPS results of the reused sample, the Ti 2p core-level scan showed no $Ti^{3+}$ defects (Figure S2 c), and the O 1s spectrum clearly indicated the presence of lattice oxygen (Figure S2 d).

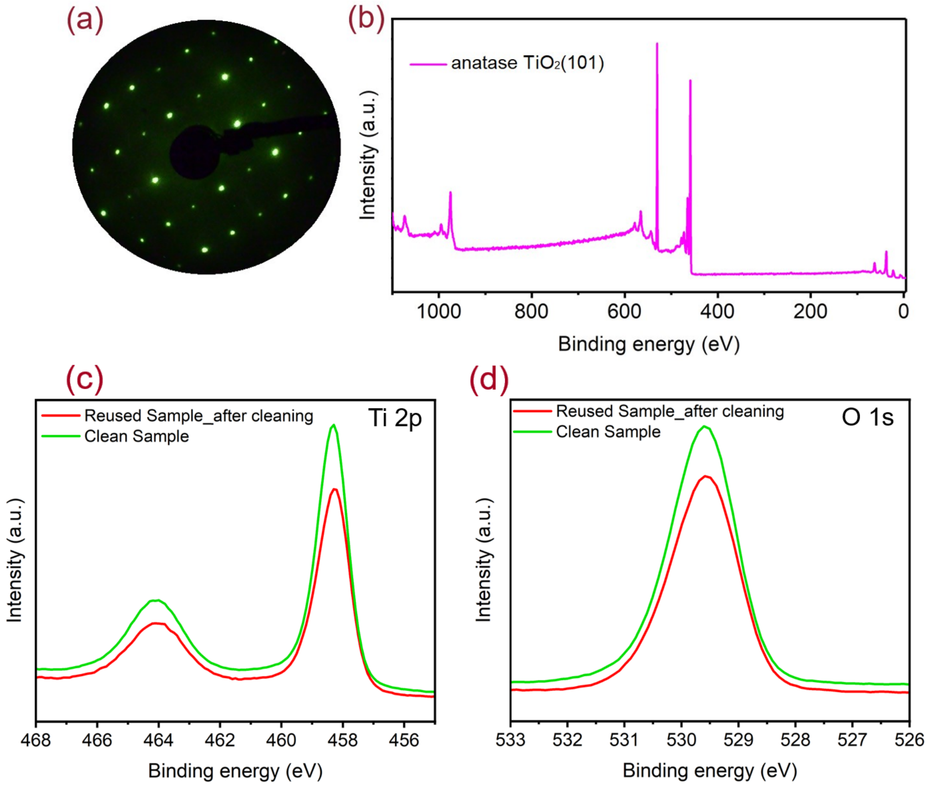


Figure S2: (a) LEED and XPS survey scan (b) of the clean anatase $TiO_2(101)$. The survey spectrum was recorded at a photon energy 1486.6 eV using a Al K$\alpha$ source. (c, d) Ti 2p and O 1s core-level scans of the clean sample and reused sample after sputtering and annealing process.

# Fluorescence Microscopy (FM)

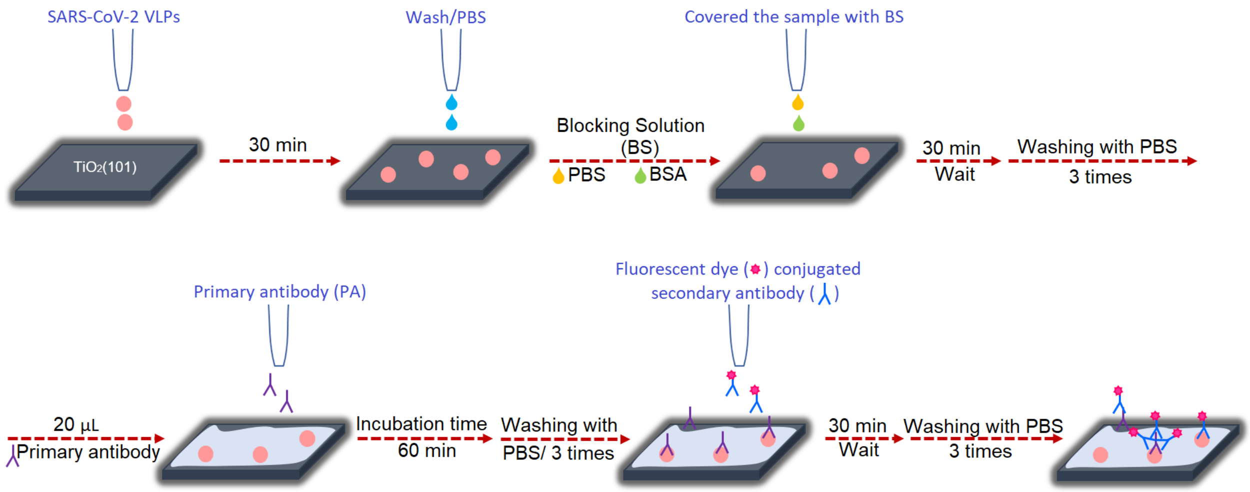


Figure S3: Sample preparation for Fluorescence Microscopy.

Table S1: Fluorescence microscopy signal for different experiments.

| Sample | FM Signal (a.u.) |
|---|---|
| $TiO_2$(101)-VLPs-PA-SA | 3407 |
| $TiO_2$(101)-VLPs-SA | 1835 |
| $TiO_2$(101)-PA-SA | 1775 |
| $TiO_2$(101)-VLPs | 1602 |

PA and SA are respectively primary and secondary antibodies.

## AFM-based Nano Infrared (Nano-IR) Spectroscopy

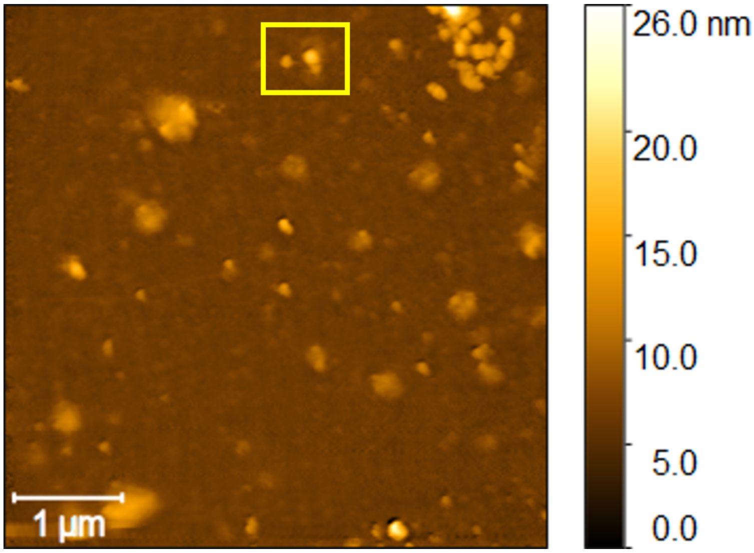


Figure S4: AFM (5.0 × 5.0 $\mu m^2$) topographic image of a larger area of VLPs adsorbed on $TiO_2$(101) surface. Marked area with yellow rectangle was selected for Nano-IR measurement.

# X-ray Photoelectron Spectroscopy (XPS)

The core-level spectra for all samples are normalized to the intensity of Ti $2p_{3/2}$ peaks of clean surface of $TiO_2(101)$. All XPS spectra were fitted by the CasaXPS software,[2] while the Shirley background and Gaussian curves were utilized. The O 1s peak (Figure S5 a) can be fitted with four compounds at binding energies 530.2 eV (lattice oxygen in $TiO_2(101)$),[3] 531.8 eV (O=C-O),[3] 532.5 eV (C=O, O=C-N)[4] and 533.6 eV (C-OH).[3]

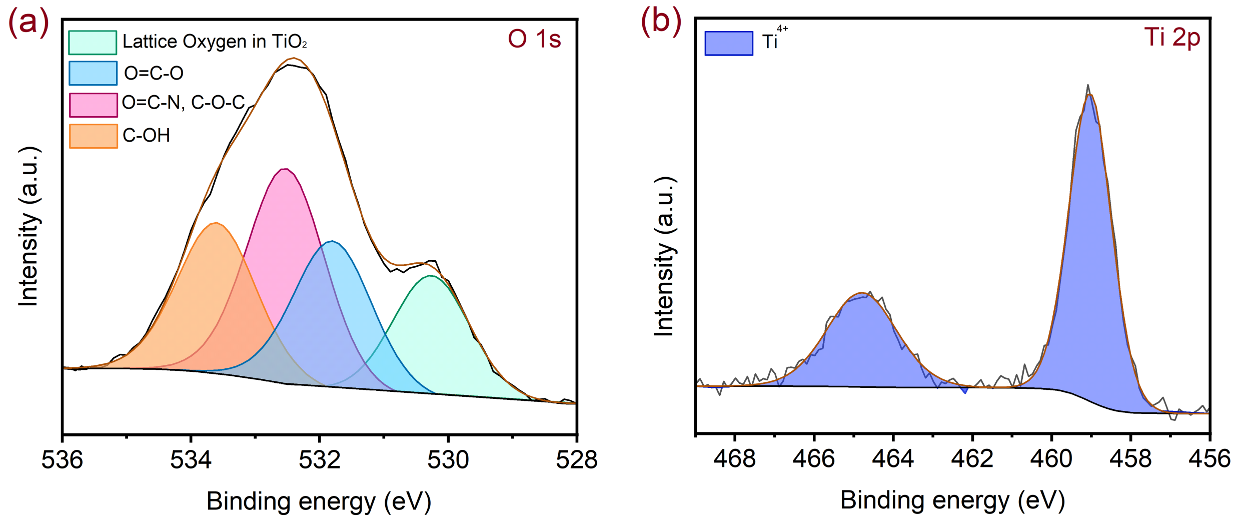


Figure S5: Deconvoluted core-level photoelectron spectra of O 1s (a) and Ti 2p (b) of adsorbed VLPs on $TiO_2(101)$ surface. The measurements were performed at a photon energy 1.486 keV.

# Computational methods

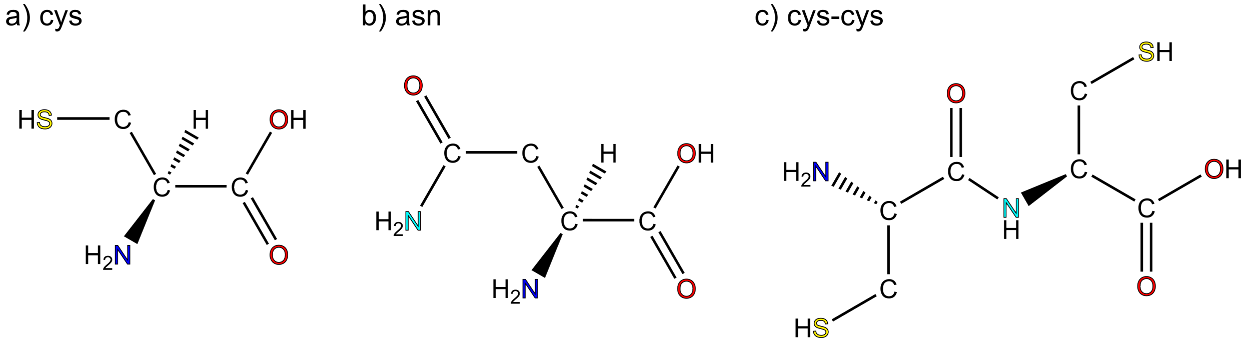


Figure S6: Sketch of isolated (a) cysteine and (b) asparagine monopeptides and (c) cysteine dimer. Different chemical species are highlighted by different colors; in the case of N groups, amino (blue) and amide (cyan) are further distinguished.

Table S2: CLSs calculated for different N species included in our adsorbed models. The isolated cysteine dipeptide is taken as the energy reference.

| model | N species | CLS (eV) |
|---|---|---|
| cys-cys | amino | 0.0 |
| cys-cys | secondary amide | 0.0 |
| cys01 | amino | 0.5 |
| cys02 | amino | -0.3 |
| cys03 | amino | 1.1 |
| cys04 | amino | 0.3 |
| asn01 | amino | -0.1 |
| asn01 | primary amide | 0.3 |

# Grazing-incidence small-angle X-ray scattering (GISAXS)

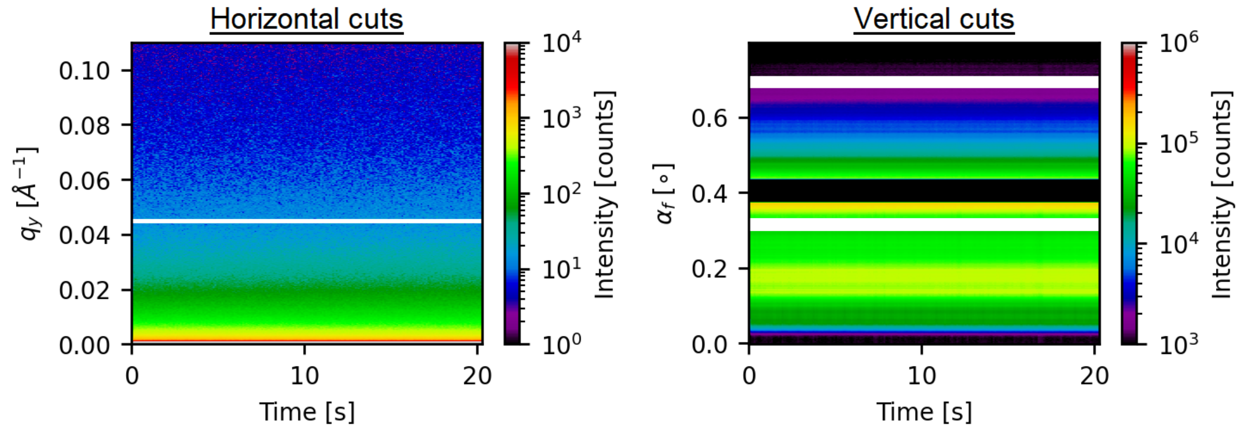


Figure S7: Horizontal and vertical cuts of beam damage scan.

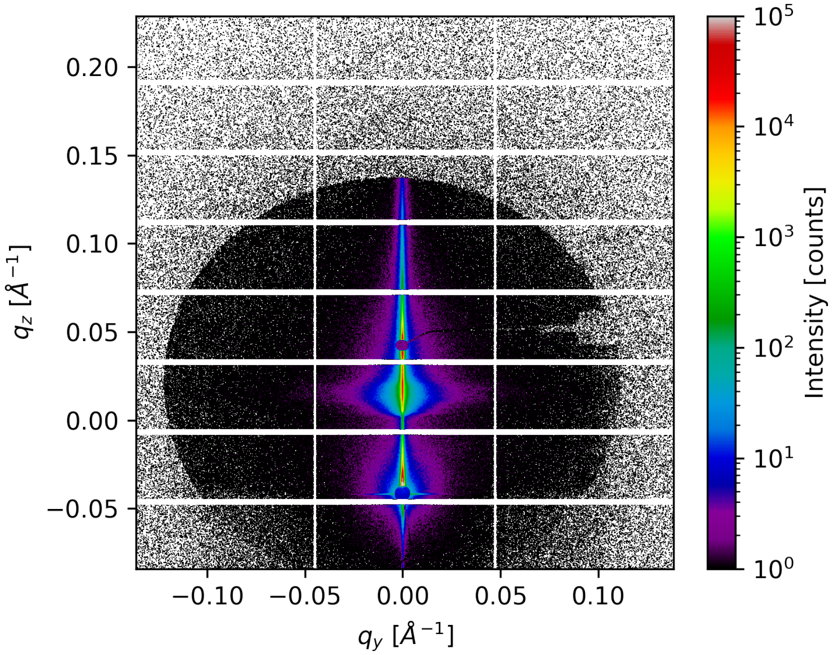


Figure S8: Exemplary raw GISAXS detector image. Note the shadowed areas due to beam stops, evacuated flight tubes and inter-modular gaps, which are present due to the experimental setup beamline P03.

Table S3: GISAXS data fitting parameters for adsorbed VLP on $TiO_2$ surface before and after UV irradiation.

| Fit parameters | VLP on $TiO_2$ before UV irradiation | VLP on $TiO_2$ after UV irradiation |
|---|---|---|
| $A_{resolution}$ (a.u.) | 9.0000 ± 0.0033 | 9.7187 ± 0.0066 |
| $w_{resolution}$ (Å$^{-1}$) | 0.001184 ± 0.000051 | 0.001184 ± 0.000051 |
| $S_{VLP}$ | (2.7000 ± 0.0092) $10^{-10}$ | (1.816 ± 0.018) $10^{-10}$ |
| $R_{VLP}$ (Å) | 251.0 ± 1.0 | 326.1 ± 1.0 |
| $PDI_{VLP}$ | 1.7585 ± 0.0047 | 1.3500 ± 0.0097 |
| $S_{surface-proteins}$ | (2.1056 ± 0.0058) $10^{-9}$ | (1.798 ± 0.014) $10^{-9}$ |
| $R_{surface-proteins}$ (Å) | 72.0 ± 1.0 | 90.0 ± 1.0 |
| $PDI_{surface-proteins}$ | 1.5006 ± 0.0053 | 1.3500 ± 0.012 |
| $S_{Byproduct}$ | (1.800 ± 0.028) $10^{-8}$ | (1.350 ± 0.011) $10^{-8}$ |
| $R_{Byproduct}$ (Å) | 12.6 ± 1.0 | 18.0 ± 1.0 |
| $PDI_{Byproduct}$ | 1.354 ± 0.016 | 1.815 ± 0.021 |
| Background (a.u.) | 0.0003973 ± 0.0000060 | 0.0007380 ± 0.0000062 |

$A_{resolution}$: height of resolution function, $w_{resolution}$: width of resolution function, $S_{VLP}$: scale factor of VLP population, $R_{VLP}$: Mean radius of VLP population, $PDI_{VLP}$: Polydispersity index (PDI) of VLP population = $\sigma_{VLP}$ / $R_{VLP}$, $\sigma_{VLP}$: Gaussian standard deviation of radii of VLP population in Å, $S_{surface-proteins}$: scale factor of Spike protein population, $R_{surface-proteins}$: Mean radius of Spike protein population, $PDI_{surface-proteins}$: Polydispersity index (PDI) of surface proteins population = $\sigma_{surface-proteins}$ / $R_{surface-proteins}$, $\sigma_{surface-proteins}$: Gaussian standard deviation of radii of surface proteins population in Å, $S_{Byproduct}$: scale factor of viral byproducts population, $R_{Byproduct}$: Mean radius of viral byproducts population, $PDI_{Byproduct}$: Polydispersity index (PDI) of viral byproducts population = $\sigma_{Byproducts}$ / $R_{Byproducts}$, $\sigma_{Byproducts}$: Gaussian standard deviation of radii of viral byproducts population in Å.

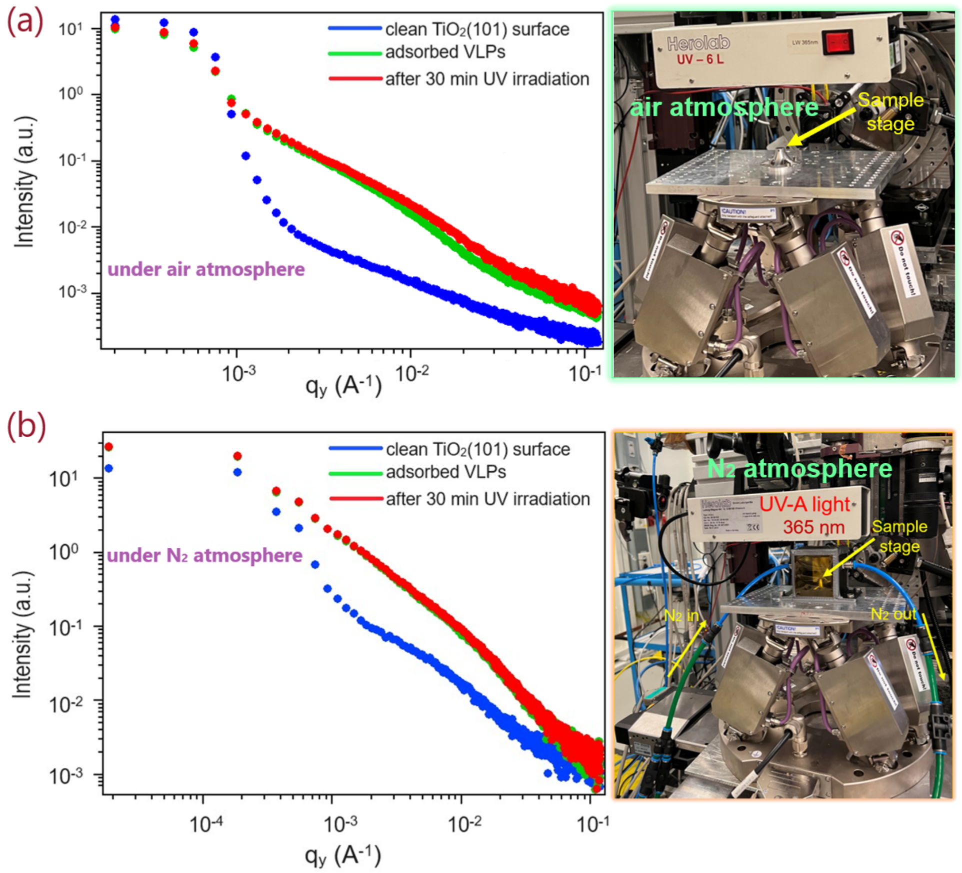


Figure S9: Left: The horizontal line cuts at the region of interest in 2D GISAXS for clean $TiO_2(101)$ surface, adsorbed VLPs and after 30 min UV-A irradiation (Wavelength: 365 nm, Intensity: 850 $\mu W/cm^2$) under air (a) and $N_2$ atmosphere (b). Right: Experimental setup at the P03 beamline for the measurements under air and $N_2$ atmosphere.

# References


(1) Stierle, A.; Keller, T. F.; Noei, H.; Vonk, V.; Roehlsberger, R. Desy nanolab. *Journal of large-scale research facilities JLSRF* **2016**, *2*, 76.

(2) Walton, J.; Wincott, P.; Fairley, N.; Carrick, A. *Peak fitting with CasaXPS: a casa pocket book*; Accolyte Science, 2010.

(3) Kohantorabi, M.; Wagstaffe, M.; Creutzburg, M.; Ugolotti, A.; Kulkarni, S.; Jeromin, A.; Krekeler, T.; Feuerherd, M.; Herrmann, A.; Ebert, G.; Protzer, U.; Guedez, G.;

Löw, C.; Thuenauer, R.; Schlueter, C.; Gloskovskii, A.; Keller, T. F.; Di Valentin, C.; Stierle, A.; Noei, H. Adsorption and Inactivation of SARS-CoV-2 on the surface of Anatase $TiO_2$(101). *ACS Appl. Mater. Interfaces.* **2023**, *15(6)*, 8770–8782.

(4) Stewart-Ornstein, J.; Hitchcock, A.; Hernandez Cruz, D.; Henklein, P.; Overhage, J.; Hilpert, K.; D.Halle J.; E.W.Hancock, R. Using Intrinsic X-ray Absorption Spectral Differences To Identify and Map Peptides and Proteins. *J. Phys. Chem. B* **2007**, *111(26)*, 7691–7699.
